# Natural variation reveals that *OsSAP16* controls low-temperature germination in rice

**DOI:** 10.1093/jxb/erx413

**Published:** 2017-12-11

**Authors:** Xiang Wang, Baohong Zou, Qiaolin Shao, Yongmei Cui, Shan Lu, Yan Zhang, Quansheng Huang, Ji Huang, Jian Hua

**Affiliations:** 1 State Key Laboratory of Crop Genetics and Germplasm Enhancement, Nanjing Agricultural University, Nanjing, China; 2 Institute of Nuclear and Biological Technology, Xinjiang Academy of Agricultural Sciences, Urumqi, China; 3 Plant Biology Section, School of Integrated Plant Science, Cornell University, Ithaca, NY, USA

**Keywords:** Gene expression, germination, rice, GWAS, low temperature, natural variation, *OsSAP16*

## Abstract

Low temperature affects seed germination in plants, and low-temperature germination (LTG) is an important agronomic trait. Natural variation of LTG has been reported in rice, but the molecular basis for this variation is largely unknown. Here we report the phenotypic analysis of LTG in 187 rice natural accessions and a genome-wide association study (GWAS) of LTG in this collection. A total of 53 quantitative trait loci (QTLs) were found to be associated with LTG, of which 20 were located in previously reported QTLs. We further identified *Stress-Associated Protein 16* (*OsSAP16*), coding for a zinc-finger domain protein, as a causal gene for one of the major LTG QTLs. Loss of *OsSAP16* function reduces germination while greater expression of *OsSAP16* enhances germination at low temperature. In addition, accessions with extremely high and low LTG values have correspondingly high and low *OsSAP16* expression at low temperatures, suggesting that variation in expression of the *OsSAP16* gene contributes to LTG variation. As the first case of identification of an LTG gene through GWAS, this study indicates that GWAS of natural accessions is an effective strategy in genetically dissecting LTG processes and gaining molecular understanding of low-temperature response and germination.

## Teaser

A genome-wide-association study of rice natural varieties identifies a stress-associated protein, OsSAP16, as a positive regulator of low-temperature germination in rice.

## Introduction

Low temperature often negatively impacts plant germination. Because of its tropical and subtropical origin, rice is more sensitive to low temperature than some other cereal crops, such as wheat and barley (Okuno, 2004). Low temperature leads to low germination rates, germination delay, low seedling vigor, decomposition of seedlings, and ultimately great reductions in yield. Low-temperature sensitivity at the germination stage is a challenge for rice cultivation, especially given that the direct seeding method is becoming a popular practice in many temperate Asian countries (Iwata *et al.*, 2010). Therefore, it is critical to understand the genetic basis of low-temperature germination (LTG) for breeding rice that is cold tolerant at the germination stage.

LTG is a complex trait and it varies greatly among rice cultivars. Over 30 LTG-related quantitative trait loci (QTLs) were detected through biparental mapping using a pair of rice cultivars (Chen *et al.*, 2006; Fujino *et al.*, 2004; Han *et al.*, 2006; Jiang *et al.*, 2017; Li *et al.*, 2013; Pan *et al.*, 2015) (see Supplementary Table S1 at *JXB* online). Among these, only one QTL, *qLTG-3-1*, has an identified causal gene, which encodes a protein with unknown function (Fujino *et al.*, 2008). The expression pattern of *qLTG-3-1* suggests that its protein product may function to weaken tissues covering the embryo during germination (Fujino *et al.*, 2008).

Currently, most of the QTLs identified for LTG were based on the segregating populations from biparental crosses. QTLs identified from different pairs of parents do not overlap in most cases, indicating the existence of more QTLs in natural populations. Recently, genome-wide association study (GWAS) based on linkage disequilibrium (LD) has been employed as an alternative strategy to dissect complex trait loci in plants, including LTG and cold tolerance at the seedling stage (Angelovici *et al.*, 2017; Atwell *et al.*, 2010; Butardo *et al.*, 2017; Digel *et al.*, 2016; Gonzalez-Jorge *et al.*, 2016; Huang *et al.*, 2013; Lv *et al.*, 2016; Shakiba *et al.*, 2017; Si *et al.*, 2016; Wang *et al.*, 2016; Zhang *et al.*, 2016; Zhao *et al.*, 2011). High-density single nucleotide polymorphisms (SNPs) are becoming more readily available owing to the development of sequencing technology, and the genomic information that they provide is expected to bridge the gap between QTLs and candidate genes (Kooke *et al.*, 2016; Mao *et al.*, 2015; Si *et al.*, 2016). Rice is a selfing plant and therefore has a modest rate of decay of LD. LD in rice will limit mapping resolution to a length of ~200 kb, which usually contains 10–20 genes (Huang *et al.*, 2012). GWAS has been successfully used to detect many QTLs and genes related to morphology and development (Zhao *et al.*, 2011), stress tolerance (Famoso *et al.*, 2011), grain size (Duan *et al.*, 2017; Si *et al.*, 2016), panicle traits (Crowell *et al.*, 2016), and many other agronomic traits (Yano *et al.*, 2016).

A rice diversity panel (RDP1) of 413 accessions collected from 82 countries has been assembled for the GWAS of various traits in rice (Zhao *et al.*, 2011). It comprises five major subpopulations: *tropical japonica* (*trj*), *temperate japonica* (*tej*), *aromatic* (*aro*), *aus* (*aus*), and *indica* (*ind*) (Eizenga *et al.*, 2014). Thirty-four traits were initially assessed on this panel using a dataset containing 44000 (44k) SNPs, and dozens of QTLs were identified (Zhao *et al.*, 2011). Further study of aluminum tolerance in subpopulations of this panel identified *Nramp aluminum transporter* (*Nrat1*) as a candidate gene for aluminum tolerance (Famoso *et al.*, 2011). More recently, a higher-density dataset of 700000 (700k) SNPs was developed for this panel, and GWAS of grain size phenotypes using the 700k SNPs on the same panel detected more QTLs than with the 44k SNPs (McCouch *et al.*, 2016). Using this panel, 49 panicle phenotypes in 242 tropical rice accessions were analyzed by GWAS with the 700k dataset; 10 genes within the QTLs identified were in pathways known to regulate plant architecture and are therefore likely to be candidate genes (Crowell *et al.*, 2016). In addition, GWAS has been applied to chilling tolerance: based on the RDP1, 67 chilling tolerance QTLs were detected in young seedlings, among which 56 had not been previously reported (Wang *et al.*, 2016). Eighteen QTLs were detected at the germination stage and 31 at the reproductive stage (Shakiba *et al.*, 2017). Therefore, GWAS using high-density SNPs of the RDP1 holds promise in dissecting the genetic architecture of a variety of traits in rice.

In this study, we conducted a GWAS on a portion of RDP1 and detected over 50 QTLs associated with LTG. We further identified *Stress-Associated Protein 16* (*OsSAP16*), coding for a zinc finger protein, as a causal gene for one QTL associated with LTG. This study sets the stage for further identification of genes important for LTG in rice.

## Materials and methods

### Plant materials

The RDP1 plants were propagated in an experimental field in Nanjing, China; harvested seeds were stored for 3 months at room temperature to break dormancy. Some accessions were not included in the GWAS study because they became infected or were lost in the field during propagation. Accessions that have deep dormancy were also not included because they would fall out of the normal range of the germination assay. Accessions in the *aro* subpopulation were also removed because of the small sample size and its complex admixed background (Zhao *et al.*, 2011).

### Assay of low-temperature germination

Germination was defined by the emergence of the coleoptile from the seed (Fujino *et al.*, 2008; Fujino *et al.*, 2004). Thirty plump and healthy seeds per line were placed on two layers of filter paper soaked with water in 9 cm Petri dishes and incubated in a growth chamber at 10°C, 12°C, 15°C, or 26°C under 12 h light/12 h day conditions. The number of germinated seeds was counted every day.

### Genome-wide association study

The genotyping data for the accessions are available at https://ricediversity.org/data/index.cfm (McCouch *et al.*, 2016; Zhao *et al.*, 2011). The GWAS was based on the compressed mixed linear model (Zhang *et al.*, 2010) and was conducted using the R package Genomic Association and Prediction Integrated Tool (Lipka *et al.*, 2012). Population structure was controlled by the top three principal components and a kinship matrix calculated according to VanRaden (2008). Manhattan plots were drawn by using the R package qqman. LD was calculated by using Tassel 5.2.31. The position of SNPs in *OsSAP16* and the protein structure of OsSAP16 are based on data from the MSU Rice Genome Annotation Project (http://rice.plantbiology.msu.edu).

### Gene expression analysis

For analysis of gene expression in leaves, plants were grown for 2 weeks in a chamber at 26°C and total RNA was extracted from leaves using TRIpure Reagent (Bio Teke Corporation, http://www.bioteke.com). For RNA extraction from seeds, seeds were imbibed in water for 2 days at 12°C or 26°C. Total RNA was extracted from six seeds for each accession using an RNA extraction kit (TRANSGEN BIOTECH, http://www.transgen.com.cn). First-strand cDNA synthesis was performed using HiScript II Q RT SuperMix (Vazyme, http://www.vazyme.com). An internal control was provided by a parallel analysis based on the actin gene *LOC_Os03g61970*. Three independent replicates were performed. Gene-specific primers for *OsSAP16* are shown in Supplementary Table S7.

### Generation of *OsSAP16* transgenic plants

The coding region of *OsSAP16* was amplified by PCR from wild-type Zhonghua 11 (ZH11) rice and cloned into the pCAMBIA1300s vector. This construct (pCAMBIA1300s-*OsSAP16*) was then transformed into ZH11 by *Agrobacterium* EHA105.

## Results

### LTG in natural rice accessions

**Fig. 1.**
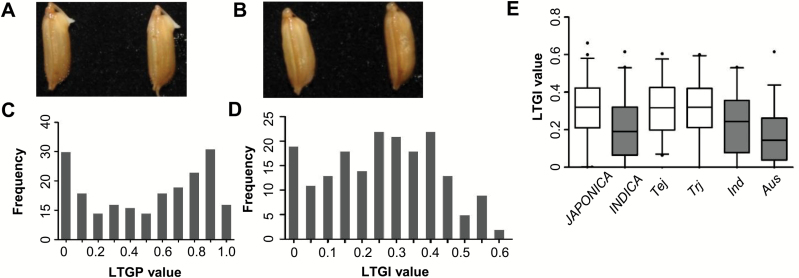
Phenotypic distribution in the 187 rice accessions. (A, B) Photographs of rice seeds showing the definition of germination (A) and no germination (B). (C, D) Frequency distribution of LTGP (C) and LTGI (D) among the 187 rice accessions. (E) Boxplot of the phenotypic variation of LTGI within varietal groups. The black horizontal lines represent the median values, the boxes represent the middle quartiles, the whiskers represent the range of data, and points represent outliers. (This figure is available in colour at *JXB* online.)

A total of 187 accessions from RDP1 (Eizenga *et al.*, 2014) were evaluated for LTG: these comprised 58 *tej*, 48 *trj*, 44 *ind*, and 37 *aus* (Supplementary Table S2). The heritability of LTG was shown to be relatively high (da Cruz *et al.*, 2006), and therefore three replicates of 30 seeds were used for LTG measurement for each accession. Germination was defined as the emergence of the coleoptile from the seed (Fig. 1A, B) (Fujino *et al.*, 2004; Fujino *et al.*, 2008). We found greater variation in the germination percentage at 12°C than at 10°C and 15°C in a randomly selected collection of 30 accessions (see Supplementary Table S3 and Supplementary Fig. S1), and therefore 12°C was chosen for the germination assessment of the 187 accessions.

Germination percentage at 12°C was analyzed from day 5 to day 13 after imbibing, when no further germination occurred. The germination percentage on day 4 in seeds maintained at 26°C was used as the value under optimal germination conditions for each respective accession, and the low-temperature germination percentage was calculated as the ratio of germination percentages at 12°C and 26°C. LTG was assessed by two parameters: low-temperature germination index (LTGI), defined as ∑(Gt/Dt), where Gt is the germination percentage on each day and Dt is the number of germination days (Ji *et al.*, 2009); and low-temperature germination potential (LTGP) defined as the germination percentage at day 10.

LTGP and LTGI in the 187 studied accessions showed large variation, ranging from 0 to 1 and 0 to 0.62, respectively (Fig. 1C, D). A correlation coefficient of 0.94 was observed between LTGP and LTGI. LTGI had a normal distribution, although it was more strongly represented in the range of 0 to 0.1 (Fig. 1D). Differences in LTGI were found among subpopulations (Fig. 1E): *tej* and *trj* had mean values of 0.32, *ind* had a lower mean value of 0.23, and *aus* had the lowest mean value of 0.18 (Supplementary Table S4). The *JAPONICA* group (consisting of *tej* and *trj*) had a mean value of 0.32, an interquartile range of 0.21–0.43, and an overall range of 0–0.60, while the *INDICA* group (consisting of *ind* and *aus*) had a mean value of 0.20, an interquartile range of 0.06–0.33, and an overall range of 0–0.62 (Fig. 1E; Supplementary Table S4). Therefore, the *JAPONICA* group is in general more cold tolerant than the *INDICA* group (*P*<0.01), which is consistent with previous observations (Lv *et al.*, 2016; Shakiba *et al.*, 2017; Wang *et al.*, 2016).

### GWAS of LTG traits in the 187 natural rice accessions

**Fig. 2.**
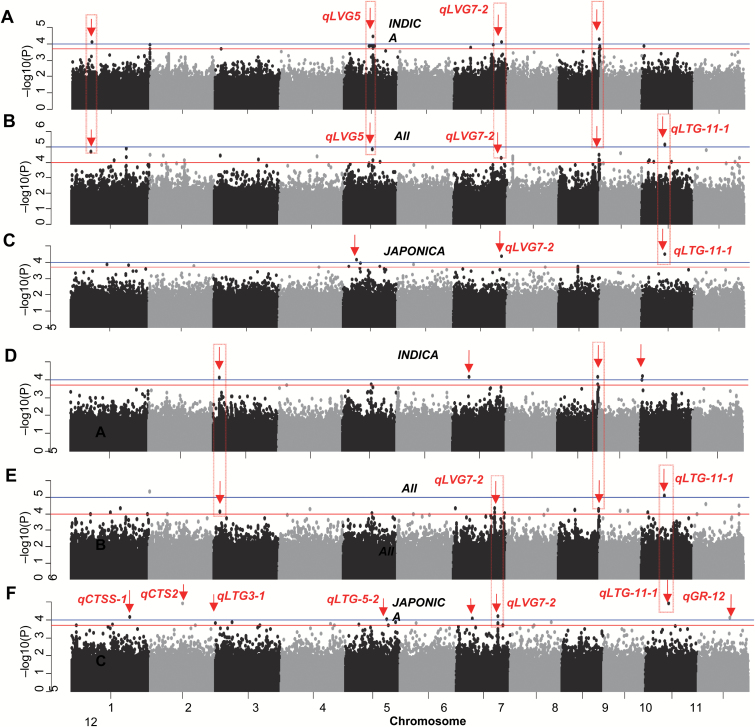
GWAS for LTG in the *ALL*, *JAPONICA*, and *INDICA* groups of accessions. Manhattan plots of LTGI analyzed in *INDICA* (A), *ALL* (B), and *JAPONICA* (C), and of LTGP in *INDICA* (D), *ALL* (E), and *JAPONICA* (F). Red arrows indicate significant SNPs detected in *JAPONICA* and *INDICA.* Boxes indicate shared significant SNPs identified by different analyses. For *JAPONICA* and *INDICA*, the blue line indicates the significance threshold set at *P*=1.0 × 10^–4^, and the red line indicates the minor significance threshold set at *P*=2 × 10^–4^. For *ALL*, the blue line indicates the significance threshold set at *P*=1.0 × 10^–5^, and the red line indicates the minor significance threshold set at *P*=1 × 10^–4^. On each plot, the X-axis represents SNP positions across the entire rice genome by chromosomes and the Y-axis is the negative logarithmic *P*-value of each SNP.

We performed GWAS on LTGP and LTGI phenotypes using a compressed mixed linear model (Lipka *et al.*, 2012; Zhang *et al.*, 2010). Because of large variation within the *JAPONICA* and *INDICA* subpopulations (Fig. 1E), GWAS was performed on the whole population as well on individual subpopulations. Manhattan plots were generated for each GWAS for easy viewing of SNPs above the significance threshold (*P*<0.0001) that was used in previous studies (Fig. 2) (Famoso *et al.*, 2011; McCouch *et al.*, 2016; Shakiba *et al.*, 2017; Wang *et al.*, 2016; Zhao *et al.*, 2011).

We detected a total of 32 LTGI-related and 22 LTGP-related QTLs when using all 187 (*ALL*) accessions (Supplementary Table S5). Among these, seven LTGI-related and eight LTGP-related QTLs were located in previously reported QTLs (Supplementary Table S5). GWAS were performed on the *INDICA* and *JAPONICA* subpopulations, Four QTLs for LTGI were found in *INDICA*; these were also detected in *ALL*, and two of them were located in the previously reported *qLVG5* and *qLVG7-2* regions (Fig. 2A, B; Supplementary Table S5). Among the three QTLs found in *JAPONICA*, one was also detected in *ALL* and was located in the previously identified *qLTG-11-1*, and another one was located in the previously reported *qLVG7-2* (Fig. 2B, C; Supplementary Table S5). For LTGP, four QTLs were detected in *INDICA*, with two also detected in *ALL* (Fig. 2D, E; Supplementary Table S5). Among seven QTLs detected in *JAPONICA*, two were also found in *ALL* and six of them co-located with previously reported QTLs of LTG or cold tolerance (Fig. 2E, F; Supplementary Table S5). At a greater *P*-value of 1.43 × 10^–4^, another QTL was detected at 30 kb away from the cloned QTL gene *qLTG-3-1* (Fig. 2F) (Fujino *et al.*, 2008), indicating the potential for identifying causal genes of QTLs revealed in GWAS.

### *OsSAP16* is a candidate causal gene for a LTG QTL

**Fig. 3.**
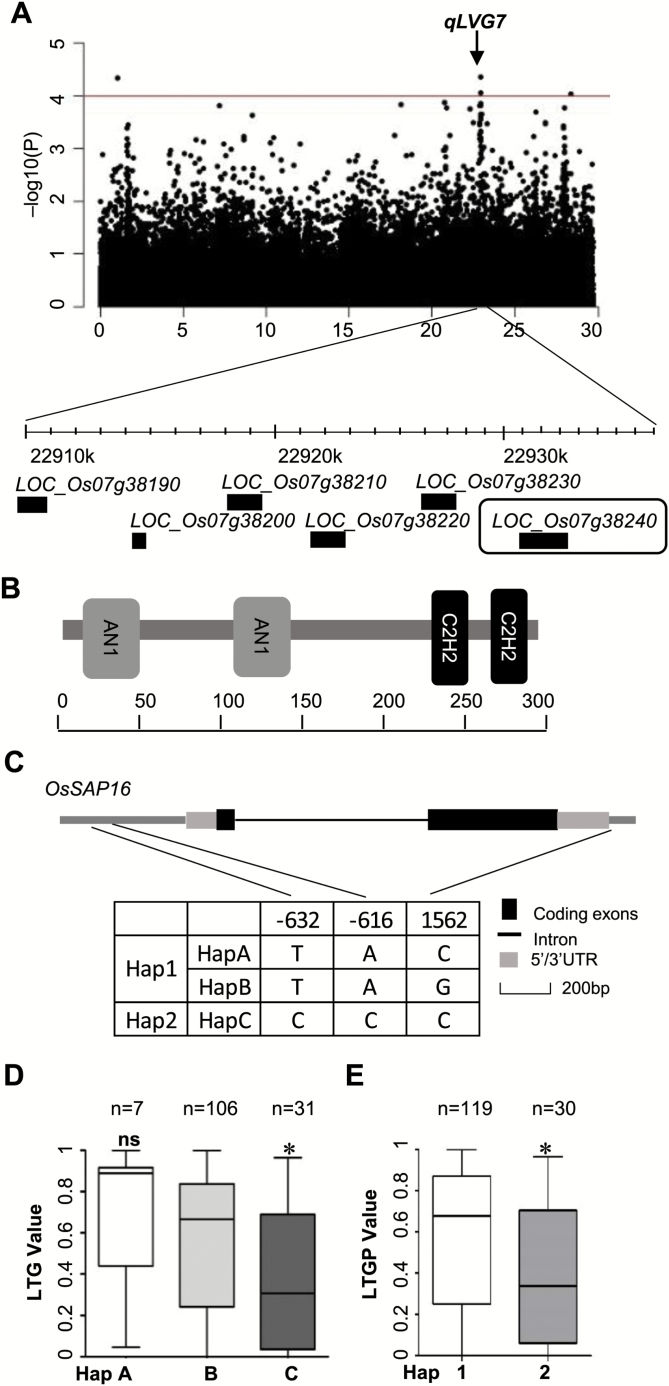
Analysis of DNA polymorphisms in the *OsSAP16* gene. (A) The genome-wide association signal and the candidate gene *LOC_Os07g38240* (*OsSAP16*) identified for LTGP on chromosome 7. (B) Protein structure of OsSAP16. OsSAP16 contains two AN1 and two C2H2 zinc finger domains. (C) Gene structure and haplotype analysis of *OsSAP16* according to the three linked (*r*^2^>0.6) SNPs shown. Positions of SNPs were defined relative to the transcription start site of the gene. (D, E) Boxplots of LTGP based on haplotypes of *OsSAP16*. n, number of the accessions with a specific haplotype. The black horizontal lines represent the median values, the boxes represent the middle quartiles, and the whiskers are the range of data. Differences of haplotypes compared with Hap B (D) and Hap 1 (E) were analyzed by Welch’s *t*-test. *P<0.05; ns, not significant.

We further investigated the LTGP QTL on chromosome 7 (Fig. 3A), because it was detected in both *ALL* and *JAPONICA* (Fig. 2E, F) and it co-localized with a previously reported QTL (Han *et al.*, 2006). It was also the only major QTL detected by GWAS on LTGP when using the 44k SNP dataset (Supplementary Fig. S2A). Because LD is 100–200 kb in rice landraces (Huang *et al.*, 2010; Si *et al.*, 2016), we examined the 200 kb region centered on the most significant SNPs in the QTL for potential causal genes and variants. The *OsSAP16* gene was identified as a good candidate for the following reasons. It is one of the 18 members of the *OsSAP* gene family coding for proteins with conserved A20/AN1-C2H2 zinc finger domains (Fig. 3B) (Vij and Tyagi, 2006). Expression of some *OsSAP* genes is responsive to abiotic stresses, including cold, salt, and dehydration, implicating these genes in stress responses (Vij and Tyagi, 2006; Wang *et al.*, 2016). The *OsSAP16* gene is highly expressed in imbibed embryo, shoot, and axillary meristems of shoots (Supplementary Fig. S3), and it is up-regulated both during germination and by cold treatment (GENEVESTIGATOR database; Supplementary Fig. S4). Numerous nucleotide sequence variation are found in the *OsSAP16* gene among the 2945 rice varieties for which whole-genome sequence information is available (Supplementary Fig. S5) (Alexandrov *et al.*, 2015). In the high-quality 700k SNP dataset, six SNPs were found in exons, among which only one, at 2036 bp 3ʹ to the transcription start site, is a non-synonymous change; the other five do not cause changes in amino acid sequences (Supplementary Fig. S6A). This missense variant is unlikely to be the causal variant because it has a very low allele frequency at 0.07 and does not correlate with LTG variation (Supplementary Fig. S6B). In addition, four SNPs (at positions –1977, –1459, –632, and –616 relative to the transcription start site) were located within 2 kb 5ʹ to the transcription start site, and three (at 2448, 2485, and 2562) were 3ʹ to the translation stop codon (Supplementary Fig. S6A). LD was calculated for these SNPs, and one linkage was found for three polymorphisms, located at –632, –616, and 2562 bp relative to the transcription start site (Fig. 3C; Supplementary Fig. S6C). When the 187 rice varieties were grouped into haplotypes A, B, and C based on these three polymorphisms, a correlation was found between these haplotypes and LTGP. Haplotype B, forming the largest group of accessions, had an average LTGP value of approximately 0.55, while haplotypes A and C had LTG values of 0.68 and 0.38, respectively (Fig.3C, D). Therefore, variation of *OsSAP16* may contribute to the variation in LTGP.

### **OsSAP16** regulates LTG

**Fig. 4.**
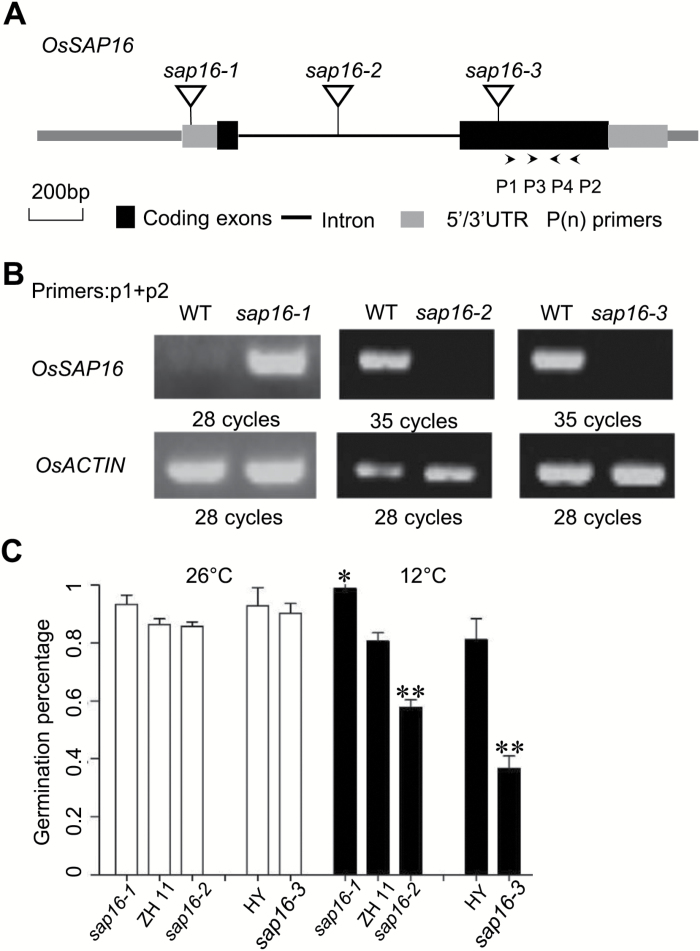
Identification of *OsSAP16* as the causal gene for a LTG QTL. (A) Schematic presentation of the gene structure of *OsSAP16* and T-DNA insertion sites in three *OsSAP16* mutants (indicated by arrowheads). P1 and P2, and P3 and P4, are two pairs of primers used to amplify *OsSAP16* cDNA for semiquantitative PCR analysis. (B) Semiquantitative RT-PCR analysis of *OsSAP16* expression in mutants. The *OsACTIN* gene was used as a normalization control. Shown are the products from 28 cycles of amplification for *OsACTIN* in all samples, 28 cycles of *OsSAP16* in *sap16-1*, and 35 cycles of *OsSAP16* for *sap16-2* and *sap16-3*. (C) Germination of wild-type Zhonghua11 (ZH11), Hwayoung (HY), and the three T-DNA insertion mutants of *OsSAP16* under 12 °C and 26 °C growth conditions. *sap16-1* in ZH11 is an overexpression mutant, and *sap16-2* in ZH11 and *sap16-3* in HY are loss-of-function mutants. Seeds were incubated for 3 days at 26 °C and for 11 days at 12 °C. Data are the means±SD from three replicates. Differences between mutants and wild types were analyzed by Welch’s *t*-test. *P<0.05, **P<0.01.

To determine whether or not *OsSAP16* is involved in LTG, we obtained one T-DNA insertion line (*sap16-1*) from Pohang University of Science and Technology and two T-DNA insertion lines (*sap16-2* and *sap16-3*) from Huazhong Agricultural University (Yi and An, 2013; Zhang *et al.*, 2006). PCR analysis confirmed T-DNA insertions in the 5ʹ untranslated region, the first intron, and the second exon in *sap16-1*, *sap16-2*, and *sap16-3*, respectively (Fig. 4A). Semiquantitative RT-PCR was then carried out to determine the expression level of *OsSAP16* in the three mutant lines. The *sap16-2* and *sap16-3* mutants had no detectable *OsSAP16* transcript, and therefore are loss-of-function mutants. In contrast, the *sap16-1* mutant, which contains a T-DNA insertion in the promoter region, showed higher expression than the wild-type ZH11, and therefore is an overexpression mutant of *OsSAP16* (Fig. 4B).

We subjected seeds of these three *OsSAP16* mutants and their respective wild-type accessions to germination under normal (26°C) and cold (12°C) temperatures. The germination percentages of the mutants were similar to that of the wild-type plants at 26°C, but not at 12°C. LTG decreased significantly from 0.81 in wild-type ZH11 to 0.58 in *sap16-2*, and from 0.81 in wild-type Hwayoung to 0.37 in *sap16-3*. In contrast, the LTG increased significantly from 0.8 in wild-type ZH11 to 1.0 in *sap16-1* (Fig. 4C). We also generated *OsSAP16* overexpressing lines and found that, similar to the *OsSAP16* higher expressor *sap16-1*, the three independent *OsSAP16* overexpressing lines had higher LTG than the wild-type ZH11 (Supplementary Fig. S7). These data indicate that *OsSAP16* is an important regulator of low-temperature germination and its expression level is correlated with LTG.

### Correlation of expression level of *OsSAP16* and LTG in natural accessions

**Fig. 5.**
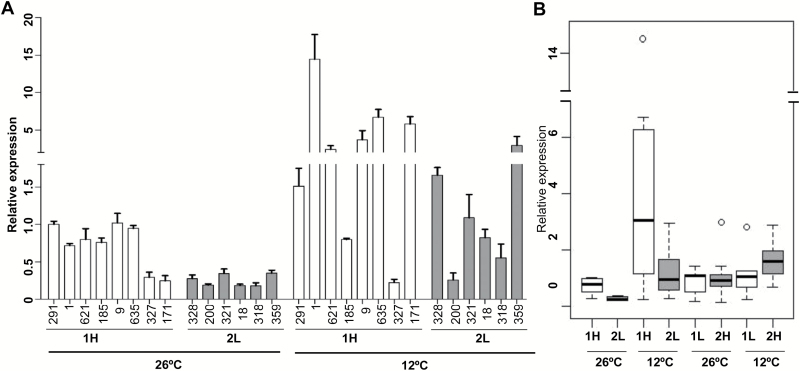
Natural variation in gene expression contributes to LTG variation. (A) Relative expression levels of *OsSAP16* in imbibed seeds of high LTG with Hap1 (1H) and low LTG with Hap2 (2L) accessions after 2 days in the dark at 12°C and 26°C, respectively. Only the number part of the accession name of RDP1 is shown, e.g. 291 represents NSFTV_291. All data in the graph are means±SD of three replicates. (B) Boxplots of the relative gene expression in different combinations of LTG and haplotypes under normal (26°C) and low-temperature (12°C) growth conditions. 1L, Hap1 with low LTG; 2H, Hap2 with high LTG.

Because the two most linked SNPs located in the *OsSAP16* promoter are correlated with LTG variation, we investigated the expression levels of these two haplotypes of *OsSAP16*, which we named Hap1 (TA) and Hap2 (CC), in different rice varieties (Fig. 3C, E). Eight varieties with Hap1 and high LTG (>0.90), referred to as 1H, and six varieties with Hap2 and low LTG (<0.10), referred to as 2L, were selected for expression analysis in seeds imbibed in water for 2 days at 12°C and 26°C. Cold induction of *OsSAP16* was observed in all but three accessions during germination (Fig. 5A). Cold induction was also reported at the seedling stage in accessions of LTH, IR29, and MH63, which were not included in the RDP1 (Supplementary Fig. S4). The 1H accessions in general had higher expression of *OsSAP16* than the 2L accessions at both temperatures. At 26°C, six of the eight 1H accessions had a relative expression level above 0.7, while all six 2L accessions had a relative expression of ~0.25. At 12°C, five of the eight 1H accessions had a relative expression above 2.5, while only one of the six 2L accessions reached a relative expression level of 2.5 (Fig. 5A).

We then examined accessions with the less common combinations of haplotypes and LTG values, that is, Hap1 with low LTG (1L) and Hap2 with high LTG (2H). Expression of *OsSAP16* was analyzed in seeds of seven 1L and nine 2H accessions imbibed with water for 2 days at 12°C and 26°C (Supplementary Fig. S8). No overall difference in *OsSAP16* expression was observed between the 1L and 2H accessions at 26°C, but the majority (six of nine) of the 2H accessions showed induction at 12°C *versus* 26°C, while the majority (five of seven) of the 1L accessions did not (Supplementary Fig. S8). Therefore, high LTG is associated with higher expression of *OsSAP16*, as was observed when comparing 1H and 2L. However, the association of expression level with the haplotypes is more complex. For Hap2, cold induction was seen for all accessions regardless of their LTG values. Relatively low expression at normal temperature was associated with low LTG, and relatively high expression at normal temperature was associated with high LTG. For Hap1 accessions, expression at normal temperature was similar, regardless of LTG value. However, induction by low temperature was associated with high LTG, and lack of induction was associated with low LTG (Fig. 5B).

These results suggest that variation in genes other than Os*SAP16* also contribute to rice LTG variation. Nevertheless, this study indicates that variation in the expression of *OsSAP16* is a major contributor to LTG variation and that the two haplotypes are associated with differences in gene expression and phenotypic variation.

## Discussion

LTG is a complex trait, as is evident from many QTLs identified from previous studies using biparental mapping. Here, we investigated LTG in nearly 200 rice natural accessions within the RDP1 by means of GWAS. A total of 53 distinct QTLs were identified, of which 20 were located in the previously reported QTLs (Supplementary Table S6). This overlap with previous findings not only validates the power of GWAS in identifying LTG QTLs, but also suggests that the RDP1 captures natural variation in the previously studied accessions. New QTLs identified in this study indicate that more genes contribute to variation in LTG in RDP1 than previously detected.

We examined both LTGP and LTGI for the LTG phenotype. Nine LTG-related QTLs were shared between LTGP and LTGI (Supplementary Table S5), and the correlation coefficient between them was very high at 0.94. LTGI measures the speed and uniformity of germination, while LTGP measures germination speed by the 10th day. The high correlation between these two parameter might be due to the fact that they both reflect many factors contributing to germination speed under low temperature. More QTLs were detected in LTGI than LTGP (32 *versus* 22) (Supplementary Table S5); this might be due to the approximately normal distribution for LTGI, which gives more detection power in GWAS.

We found that the *JAPONICA* subpopulation (*tej* and *trj*) in general has a higher LTGI value than *INDICA* (*ind* and *aus*) (Fig. 1E). Indeed, most of the alleles with increased LTG identified in previous studies belong to *JAPONICA* (Chen *et al.*, 2006; Fujino *et al.*, 2008; Fujino *et al.*, 2004; Han *et al.*, 2006; Jiang *et al.*, 2006; Li *et al.*, 2013). This finding is consistent with the notion that *JAPONICA* is adapted to the low temperatures that occur at high latitudes and higher elevations, while *INDICA* is adapted to low-latitude regions (Lv *et al.*, 2016). The difference between *JAPONICA* and *INDICA* could have resulted from environmental selection or human domestication (McCouch, 2004). This finding also suggests that *JAPONICA* varieties contain alleles that would be useful to enhance the LTG traits of *INDICA*. Within *JAPONICA*, no significant difference in LTGI was observed between *tej* and *trj* (Fig. 1E), which is consistent with the idea that *tej* and *trj* are closely related and have a lower genetic diversity (Ni *et al.*, 2002). In contrast, *ind* and *aus* in *INDICA* showed significant differences in LTGI (Fig. 1E), consistent with a high genetic diversity in this subpopulation (Zhao *et al.*, 2011).

In addition, QTLs identified within the *INDICA* and *JAPONICA* subpopulations were often not shared between the two subpopulations (Fig. 2). This could be the result of non-overlapping genetic variation between *JAPONICA* and *INDICA*, supporting the earlier notion that *JAPONICA* and *INDICA* were domesticated independently from different geographical and ecological locations (Kovach *et al.*, 2007). It is also possible that shared QTLs do exist but were not detected due to the small sample size used in this study. In this case, allele frequency would still vary greatly between the two subpopulations, suggesting a limited amount of introgression between the subpopulations.

This study identified *OsSAP16* as a regulator of LTG. The expression level of *OsSAP16* is correlated with the level of LTG. *OsSAP16* is up-regulated during germination and is induced by cold (Supplementary Fig. S4). An *OsSAP16* high expression allele and three independent transgenic *OsSAP16* overexpressing lines exhibited higher LTG, while loss-of-function mutant lines exhibited lower LTG than the wild type (Fig. 4C). The causal variation in the *OsSAP16* gene responsible for LTG variation in the natural population are not yet defined. Interestingly, the level of expression of *OsSAP16* in imbibed seeds at low temperature is largely correlated with LTG in the rice natural accessions studied here: high expression is associated with high LTG and low expression is associated with low LTG (Fig. 5B). We have not determined the exact SNP(s) responsible for the variation of *OsSAP16* expression or LTG, but two linked SNPs located in the *OsSAP16* promoter region are associated with LTG. Hap1 was often associated with high LTG (H1) and high expression at both normal and cold temperatures. In rare cases where Hap1 was associated with a low LTG (L1), *OsSAP16* was not induced by cold. Hap2 was often associated with low LTG (L2) and low *OsSAP16* expression, the exception being when it showed high expression at normal temperature. Therefore, these SNPs (or their associated SNPs) are likely major determinants of the expression level of *OsSAP16*. Nevertheless, as LTG is a complex trait, both the induction and the basal level of *OsSAP16* are also regulated by other genes, perhaps located in other QTLs, which could explain the above-mentioned exceptions to the correlation with *OsSAP16* expression.

*OsSAP16* encodes a stress-associated protein containing two AN1-C2H2 zinc finger domains. In human beings, A20, a zinc finger protein with a similar domain structure, acts as a ubiquitin ligase and targets the transcription factor NF-kappa B for degradation in innate immunity (Heyninck and Beyaert, 2005). In plants, expression of the *SAP* genes is responsive to multiple abiotic and biotic stresses (Tyagi *et al.*, 2014). Overexpression of *OsSAP1* enhances disease resistance to bacterial pathogens and expression of defense genes (Tyagi *et al.*, 2014). An activation tagging mutant screen found that two mutant lines defective in water use efficiency had a T-DNA insertion close to *sap16-1*, which resulted in overexpression of *OsSAP16* (Wang *et al.*, 2016). These two mutants had limited CO_2_ assimilation, increased expression of stress-response genes, and reduced growth. With regard to the *sap16-1* allele studied here, the *OsSAP16* gene is overexpressed but no obvious growth defect is observed (Supplementary Fig. S9). Therefore, it is yet to be determined whether or not the phenotypes seen in the activation tagging mutants are due to the overexpression of *OsSAP16* or other genes nearby.

*OsSAP16* is one of the very few cloned genes that regulate germination at low temperature in rice. The previously identified LTG gene *qLTG-3-1* gene is likely involved in germination in general but is not specific to low temperature (Fujino *et al.*, 2008). *OsSAP16* is induced by low temperature and by germination, and its expression level determines LTG. The *OsSAP16* loss-of-function mutants studied here have low germination at low temperature but wild-type germination at normal temperature. These observations indicate that *OsSAP16* is a critical regulator of low-temperature germination. In addition, haplotypes associated with high expression levels of *OsSAP16* provide a genetic resource for improving germination at low temperature. How *OsSAP16* regulates LTG is still unknown. During germination, there are large changes in RNA transcripts and proteins as seeds transit from dormancy to the growth phase. Perhaps *OsSAP16* regulates the abundance of proteins that need to be degraded during germination; up-regulation of *OsSAP16* would be needed for the efficient degradation of such proteins at low temperatures, when the germination process is slowed down. Future identification of target proteins of *OsSAP16* will further our understanding of low-temperature adaptation and germination control.

## Supplementary data

Supplementary data are available at *JXB* online.

Table S1. Previously identified QTLs of LTG.

Table S2. Information on 187 accessions and phenotypic value of LTGP and LTGI.

Table S3. LTGP of 30 randomly selected accessions under 15 °C and 12 °C.

Table S4. Maximum, minimum, and average values of LTGI within varietal groups.

Table S5. Significant SNPs of LTGP and LTGI based on the 700k dataset and the 44k dataset.

Table S6. Different SNPs associated with LTG.

Table S7. Information on the primers used in this study.

Fig. S1. Frequency distribution of LTGP of 30 randomly selected accessions under 15°C and 12°C.

Fig. S2. Manhattan plots of GWAS on LTGP and LTGI using the 44k dataset.

Fig. S3. Relative expression of *OsSAP16* in different cell types and plant tissues of rice.

Fig. S4. Gene expression profiles of *OsSAP16* from GENEVESTIGATOR.

Fig. S5. SNPs located in *OsSAP16* across the 2945 rice varieties based on IRIC.

Fig. S6. DNA polymorphism in the *OsSAP16* gene present in the 700k dataset.

Fig. S7. Phenotypes of *OsSAP16* overexpressing lines.

Fig. S8. Relative expressions of *OsSAP16* in imbibed seeds of various accessions after 2 days in the dark at 12°C and 26°C respectively.

Fig. S9. *OsSAP16* mutants and the wild type grown at 26°C for 14 days in 0.35% agar.

Supplementary Tables and Figures

## Abbreviations:

aro: *aromatic*
GWAS: genome-wide association study
HY: Hwayoung
ind: , *indica*
LD: linkage disequilibrium
LTG: low-temperature germination
LTGP: low-temperature germination potential
LTGI: low-temperature germination index
QTL: quantitative trait locus
RDP1: rice diversity panel
SAP: stress association protein
SNP: single nucleotide polymorphism
tej: *temperate japonica*
trj: *tropical japonica*
ZH11: Zhonghua 11.
