# Supplementary material for: Natural variation reveals that OsSAP16 controls low-temperature germination in rice
Source: J Exp Bot. 2017 Dec 11;69(3):413–21. doi: 10.1093/jxb/erx413 (PMC5853544; doi:10.1093/jxb/erx413)
Supplement: Supplementary Tables and Figures [file erx413_suppl_supplementary_tables_and_figures.pdf]

Supplemental Table S1. Previous identified QTLs of LTG.

QTL	Chromosome	Trait	Reference
qLTG3-1	3	GR for 7d (15 ºC)	Fujino et al. 2004
qLTG3-2	3		
qLTG4	4		
qLVG2	2	vigor of germination (14 ºC)	Han et al. 2006
qLVG3	3		
qLVG5	5		
qLVG6	6		
qLVG7-1	7		
qLVG7-2	7		
qLVG8	8		
qLVG11	11		
qLTG-9	9	GR for 4,5d (15 ºC)	Li et al. 2013
qLTG-7	7		
qLTG-12	12		
qLTG-5-1	5	GR for 7-15d (15 ºC)	Jiang et al. 2006
qLTG-5-2	5		
qLTG-5-3	5		
qLTG-4	4		
qLTG-3	3		
qLTG-9	9		
qLTG-10	10		
qLTG-11-1	11		
qLTG-11-2	11		
qGR-2	2	GR for 6-10d (15 ºC)	Ji et al. 2009
qGR-3	3		
qGR-7	7		
qGR-10	10		
qGR-11	11		
qGR-12	12		
qGI-2	2	GI for 10d (15 ºC)	
qGI-7	7		
qGI-10	10		
qGI-11	11		

Supplemental Table S2. Information of 187 accessions and phenotypic value of LTGP and LTGI

ID	Plant name	groups	LTGP	LTGI
NSFTV_4	NSF-TV 4	AUS	0.2667	0.159
NSFTV_6	ARC 7229	AUS	0	0
NSFTV_13	NSF-TV 13	AUS	0.9231	0.4024
NSFTV_18	BJ 1	AUS	0.5	0.2179
NSFTV_19	Black Gora	AUS	0.3667	0.2462
NSFTV_33	Chuan 4	AUS	0.0345	0.1512
NSFTV_44	Dhala Shaitta	AUS	0.1481	0.1111
NSFTV_78	Jhona 349	AUS	0.8148	0.4188
NSFTV_85	Kasalath	AUS	0	0.0051
NSFTV_88	Khao Gaew	AUS	0	0.0168
NSFTV_105	Mehr	AUS	0.6897	0.3263
NSFTV_131	Phudugey	AUS	0.25	0.1264
NSFTV_200	P 737	AUS	0.3077	0.2041
NSFTV_228	CA 902/B/2/1	AUS	0.75	0.4038
NSFTV_261	Shim Balte	AUS	0.7586	0.3263
NSFTV_262	Halwa Gose Red	AUS	0	0
NSFTV_276	Kaukau	AUS	0.0333	0.0308
NSFTV_312	Aswina 330	AUS	0.0714	0.1126
NSFTV_314	CTG 1516	AUS	0.1724	0.1194
NSFTV_316	DD 62	AUS	0.5313	0.2524
NSFTV_317	DJ 123	AUS	0.1724	0.1406
NSFTV_319	DK 12	AUS	0	0.0769
NSFTV_320	DM 43	AUS	0	0.0641
NSFTV_321	DM 56	AUS	0.3929	0.239
NSFTV_322	DM 59	AUS	0.5357	0.2445
NSFTV_323	DNJ 140	AUS	0	0.037
NSFTV_324	DV 123	AUS	0	0.0769
NSFTV_327	Goria	AUS	0.9333	0.4385
NSFTV_328	Jamir	AUS	0.1429	0.1429
NSFTV_330	Khao Pahk Maw	AUS	0.6087	0.2742
NSFTV_341	Shirkati	AUS	0.8846	0.429
NSFTV_359	Surjamkuhi	AUS	0	0.0385
NSFTV_360	PTB 30	AUS	0	0
NSFTV_369	Sathi	AUS	0.9643	0.6154
NSFTV_370	Coarse	AUS	0.6207	0.2626
NSFTV_372	Sufaid	AUS	0.0769	0.071
NSFTV_378	Kalubala Vee	AUS	0	0
NSFTV_3	Ai-Chino-Hong	IND	0	0.041
NSFTV_21	Byakkoku Y 5006 Seln	IND	0.4	0.2051
NSFTV_29	Chau	IND	0.4667	0.2077
NSFTV_57	NSF-TV 57	IND	0	0
NSFTV_71	IR 36	IND	0.8333	0.3154
NSFTV_72	IR 8	IND	0.8611	0.3526
NSFTV_76	Jaya	IND	0.8333	0.3821
NSFTV_90	Kiang-Chou-Chiu	IND	0.7	0.3769
NSFTV_97	Kun-Min-Tsieh-Hunan	IND	0.6333	0.2821
NSFTV_106	Ming Hui	IND	0	0.024
NSFTV_109	MTU9	IND	0.8387	0.4194
NSFTV_117	O-Luen-Cheung	IND	0.125	0.1082
NSFTV_125	Pao-Tou-Hung	IND	0	0
NSFTV_129	Peh-Kuh	IND	0	0
NSFTV_130	Peh-Kuh-Tsao-Tu	IND	0.1034	0.0345
NSFTV_132	Rathuwee	IND	0	0
NSFTV_137	RTS14	IND	0.1	0.1615

NSFTV_142	Shai-Kuh	IND	0.7778	0.3162
NSFTV_156	Taichung Native 1	IND	0.3824	0.19
NSFTV_171	ZHE 733	IND	0.931	0.5093
NSFTV_172	Zhenshan 2	IND	0.7429	0.4286
NSFTV_178	ARC 6578	IND	0.6897	0.3156
NSFTV_189	Criollo La Fria	IND	0.6207	0.2759
NSFTV_203	Radin Ebos 33	IND	0.7586	0.321
NSFTV_209	Tchibanga	IND	0.0714	0.0962
NSFTV_231	Hunan Early DwarfNo.3	IND	0.7857	0.3681
NSFTV_235	Sze Guen Zim	IND	0	0.0308
NSFTV_252	Djimoron	IND	0.0714	0.1291
NSFTV_284	IR-44595	IND	0.6	0.2872
NSFTV_298	LD 24	IND	0.069	0.0902
NSFTV_299	SML 242	IND	0.8333	0.3974
NSFTV_313	BR24	IND	0.3571	0.1621
NSFTV_318	DJ 24	IND	0.069	0.1671
NSFTV_356	JC 117	IND	0	0.0085
NSFTV_385	Nira	IND	0.1389	0.1432
NSFTV_620	Jasmine 85	IND	0.7333	0.3564
NSFTV_629	Panda	IND	0.25	0.2308
NSFTV_633	Jing 185-7	IND	0.6	0.2564
NSFTV_634	Rondo (4484-1693)	IND	0.1071	0.0742
NSFTV_616	RT0034	IND	0.7727	0.3147
NSFTV_636	Sadu Cho	IND	0	0.0571
NSFTV_642	Zhenshan 97B(ZS97B)	IND	0.8519	0.5328
NSFTV_643	Minghui 63(MH 63)	IND	0.6316	0.2733
NSFTV_644	IR64-21	IND	0.8333	0.3974
NSFTV_648	han-Huang-Zhan-2(SHZ	IND	0.9286	0.4533
NSFTV_8	Asse Y Pung	TRJ	0.7241	0.2759
NSFTV_23	Canella De Ferro	TRJ	0.8	0.3487
NSFTV_24	Carolina Gold	TRJ	0.9286	0.3826
NSFTV_25	Carolina Gold	TRJ	0	0
NSFTV_26	Carolina Gold Sel	TRJ	0.0345	0.0133
NSFTV_65	Honduras	TRJ	0.7586	0.3156
NSFTV_69	IAC 25	TRJ	0.5455	0.1678
NSFTV_73	IRAT 177	TRJ	0.6154	0.2101
NSFTV_98	L-202	TRJ	0.4444	0.2735
NSFTV_99	LAC 23	TRJ	0	0
NSFTV_108	Moroberekan	TRJ	0.0455	0.021
NSFTV_120	OS6	TRJ	0.5172	0.2387
NSFTV_135	RT 1031-69	TRJ	0.3571	0.2115
NSFTV_150	Sultani	TRJ	0.931	0.4881
NSFTV_152	T 1	TRJ	0	0.0026
NSFTV_164	Tondok	TRJ	0.2414	0.2042
NSFTV_165	Trembese	TRJ	0.9677	0.4665
NSFTV_183	Boa Vista	TRJ	0.8846	0.4586
NSFTV_185	British Honduras Creole	TRJ	1	0.565
NSFTV_195	IRAT 13	TRJ	0.8966	0.3236
NSFTV_198	Leah	TRJ	0.8519	0.3647
NSFTV_202	Pratao	TRJ	1	0.5256
NSFTV_213	WC 3397	TRJ	0.9655	0.4748
NSFTV_214	WC 4419	TRJ	0.7586	0.2653
NSFTV_215	WC 4443	TRJ	0.7667	0.2923
NSFTV_239	WAB 502-13-4-1	TRJ	0	0.0026
NSFTV_240	WAB 501-11-5-1	TRJ	0.7333	0.3359
NSFTV_285	Tox 782-20-1	TRJ	0.871	0.3573
NSFTV_308	Llanero 501	TRJ	0.8929	0.3544
NSFTV_309	Manzano	TRJ	0.92	0.4769

NSFTV_379	Wanica	TRJ	0.6786	0.2967
NSFTV_384	318	TRJ	0.5926	0.2906
NSFTV_391	Della	TRJ	0.9	0.3256
NSFTV_392	Edith	TRJ	0.2414	0.2095
NSFTV_394	Lady Wright Seln	TRJ	0.9655	0.3926
NSFTV_396	Cocodrie	TRJ	0.9032	0.4069
NSFTV_397	Cybonnet	TRJ	0.5625	0.2596
NSFTV_352	Guatemala 1021	TRJ	0.8966	0.4244
NSFTV_375	Upland	TRJ	0.5517	0.2573
NSFTV_621	LaGrue	TRJ	1	0.6
NSFTV_619	Rosemont	TRJ	0.4	0.1667
NSFTV_624	Kaybonnet	TRJ	0.9	0.3692
NSFTV_628	Jefferson	TRJ	0.5185	0.1909
NSFTV_630	Saber	TRJ	0.9	0.5538
NSFTV_625	Katy	TRJ	0.6897	0.3156
NSFTV_635	Azucena	TRJ	0.9667	0.5692
NSFTV_647	Cypress	TRJ	0.963	0.4188
NSFTV_177	68-2	TEJ	0.75	0.4066
NSFTV_158	Taipei 309	TEJ	0.8929	0.456
NSFTV_300	Sml Kapuri	TEJ	0.8846	0.4172
NSFTV_173	Nipponbare	TEJ	0.16	0.0646
NSFTV_283	Chibica	TEJ	0.7308	0.3402
NSFTV_151	Suweon	TEJ	0.8	0.3577
NSFTV_94	Koshihikari	TEJ	0.2727	0.1538
NSFTV_297	Bahia	TEJ	0.6667	0.3795
NSFTV_267	Hatsunishiki	TEJ	0.7391	0.3645
NSFTV_250	Bulgare	TEJ	0.6296	0.2991
NSFTV_311	56-122-23	TEJ	0.7059	0.3982
NSFTV_133	Rikuto Kemochi	TEJ	0.8667	0.541
NSFTV_86	Kaw Luyoeng	TEJ	0.1429	0.0769
NSFTV_1	Agostano	TEJ	1	0.5165
NSFTV_220	Azerbaidjanica	TEJ	0.3478	0.2441
NSFTV_306	WIR 3764	TEJ	0.7586	0.4005
NSFTV_62	Gyehwa 3	TEJ	0.1613	0.1663
NSFTV_186	Bul Zo	TEJ	0.24	0.1754
NSFTV_9	Baber	TEJ	0.9167	0.4904
NSFTV_295	Bombilla	TEJ	1	0.5449
NSFTV_204	Razza 77	TEJ	0.75	0.4038
NSFTV_257	Agusita	TEJ	0.8889	0.3789
NSFTV_265	Vialone	TEJ	0.4286	0.1978
NSFTV_143	Shinriki	TEJ	0.25	0.1282
NSFTV_302	WIR 3039	TEJ	0.0455	0.0804
NSFTV_115	NPE 835	TEJ	0.5455	0.2902
NSFTV_282	Triomphe Du Maroc	TEJ	0.913	0.5385
NSFTV_288	Italican Carolina	TEJ	0.8333	0.4295
NSFTV_179	Bellardone	TEJ	0.9643	0.2692
NSFTV_180	Benllokk	TEJ	0.6923	0.3254
NSFTV_301	Melanotrix	TEJ	0.44	0.2431
NSFTV_32	Chodongji	TEJ	0.9444	0.5256
NSFTV_192	Erythroceros Hokkaido	TEJ	0.6087	0.3311
NSFTV_248	Caucasica	TEJ	0.4211	0.1741
NSFTV_639	Nipponbare	TEJ	0.1538	0.2071
NSFTV_289	Lusitano	TEJ	0.1364	0.1224
NSFTV_334	Lomello	TEJ	0.7083	0.3462
NSFTV_291	Toploea 70/76	TEJ	0.9259	0.4302
NSFTV_263	Maratelli	TEJ	0.8261	0.4381
NSFTV_368	Deokjeokjodo	TEJ	0.8333	0.4615
NSFTV_233	Sung Liao 2	TEJ	0.25	0.2308

NSFTV_154	Ta Hung Ku	TEJ	0.3448	0.2414
NSFTV_296	Dosel	TEJ	0.6667	0.2917
NSFTV_365	Shirogane	TEJ	0.6429	0.2802
NSFTV_287	Zerawchanica Karatalski	TEJ	0.9	0.4231
NSFTV_113	Norin 20	TEJ	0.2727	0.1608
NSFTV_281	Patna	TEJ	0.1	0.0808
NSFTV_641	Tainung 67(TNG67)	TEJ	0	0.0077
NSFTV_247	Desvauxii	TEJ	0.9	0.3885
NSFTV_290	Amposta	TEJ	0.1579	0.166
NSFTV_157	Tainan Iku 487	TEJ	0.5	0.3077
NSFTV_118	Oro	TEJ	0.6087	0.2642
NSFTV_225	Biser 1	TEJ	0.55	0.2769
NSFTV_15	Beonjo	TEJ	0.25	0.0923
NSFTV_380	Tainan-Iku No.512	TEJ	0.7333	0.2718
NSFTV_245	Sab Ini	TEJ	0.7	0.25
NSFTV_104	Mansaku	TEJ	0.3158	0.1984
NSFTV_219	Nucleoryza	TEJ	0.85	0.4346

Supplemental Table S3. LTGP of randomly selected 30 accessions.

ID	LTGP (12ºC)	LTGP (15ºC)
NSFTV_633	0.60	0.91
NSFTV_628	0.52	0.69
NSFTV_245	0.70	0.92
NSFTV_203	0.76	0.41
NSFTV_125	0.00	0.86
NSFTV_157	0.50	0.45
NSFTV_178	0.69	0.83
NSFTV_635	0.97	0.94
NSFTV_120	0.52	0.67
NSFTV_132	0.00	0.88
NSFTV_189	0.62	0.69
NSFTV_13	0.92	0.91
NSFTV_44	0.15	0.91
NSFTV_239	0.00	1.00
NSFTV_322	0.54	0.91
NSFTV_26	0.03	0.93
NSFTV_625	0.69	0.98
NSFTV_189	0.62	0.85
NSFTV_233	0.25	0.89
NSFTV_108	0.05	0.93
NSFTV_154	0.34	0.75
NSFTV_296	0.67	0.84
NSFTV_365	0.64	0.70
NSFTV_287	0.90	0.96
NSFTV_113	0.27	0.70
NSFTV_641	0.00	0.32
NSFTV_290	0.16	0.10
NSFTV_118	0.61	0.87
NSFTV_225	0.55	0.57
NSFTV_15	0.25	0.78

Supplemental Table S4. statistic value of LTGI within varietal groups.

Groups	Max	Min	Average
JAPONICA	0.6	0	0.32
INDICA	0.62	0	0.2
Tej	0.6	0.06	0.32
Trj	0.6	0	0.31
Ind	0.53	0	0.18
Aus	0.62	0	0.23

Supplementary Table S5. Significant SNPs of LTGP, LTGI based on 700k dataset and 44k dataset.

SNP	Chromosme	position	p-value	QTL	reference	index and dataset
SNP-11.12306350.	11	12770030	7.48E-06	qLTG-11-1/qSCT11	Jiang et al. 2006/Kim et al. 2014	
SNP-1.30553988.	1	30555033	1.30E-05	qLVG-1	Chen et al. 2006	
SNP-5.15846104.	5	15903564	1.47E-05	qLVG5	Han et al. 2006	
SNP-12.6717912.	12	6719023	1.59E-05			
SNP-1.10721450.	1	10722475	1.98E-05			
SNP-10.11494085.	10	11565267	2.55E-05	qLTG-10	Jiang et al. 2006	
SNP-2.489904.	2	489905	3.05E-05			
SNP-9.22542045.	9	22542527	3.39E-05			
SNP-3.3410668.	3	3411673	3.64E-05			
SNP-2.7783346.	2	7783348	3.81E-05			
SNP-8.3108386.	8	3109384	3.97E-05			
SNP-4.20466240.	4	20638198	4.01E-05			
SNP-8.3114101.	8	3115099	4.40E-05			
SNP-1.30416673.	1	30417718	4.68E-05	qLTG-1	Chen et al. 2006	
SNP-7.26278120.	7	26279115	4.93E-05	qLVG7-2	Han et al. 2006	
SNP-12.25114399.	12	25147946	5.31E-05			LTGI/*ALL*
SNP-8.3097042.	8	3098040	6.23E-05			
SNP-9.22541472.	9	22541954	6.36E-05			
SNP-3.24464619.	3	24466541	6.37E-05			
SNP-9.22541232.	9	22541714	6.42E-05			
SNP-5.16324989.	5	16382508	7.01E-05			
SNP-2.19497594.	2	19503463	7.13E-05			
SNP-11.4219309.	11	4223408	7.27E-05	qLVG11	Han et al. 2006	
SNP-9.22844340.	9	22844822	7.34E-05			
SNP-1.23843134.	1	23844179	7.38E-05			
SNP-9.22541111.	9	22541593	7.96E-05			
SNP-9.22512729.	9	22513211	8.39E-05			
SNP-5.20140745.	5	20203273	8.87E-05			
SNP-12.22744562.	12	22778124	9.14E-05			
SNP-11.6595377.	11	6599625	9.25E-05			
SNP-11.16013664.	11	16478788	9.44E-05			
SNP-11.3673951.	11	3678050	9.48E-05			
SNP-2.489904.	2	489905	4.20E-06			
SNP-11.12306350.	11	12770030	7.83E-06	qLTG-11-1/qSCT11	Jiang et al. 2006/Kim et al. 2014	
SNP-12.6717912.	12	6719023	2.71E-05			
SNP-12.25114399.	12	25147946	3.35E-05			
SNP-7.22916319.	7	22917313	4.44E-05	qLVG7-2	Han et al. 2006	
SNP-7.1026437.	7	1027438	4.61E-05			
SNP-1.27481758.	1	27482803	4.85E-05			
SNP-9.22542045.	9	22542527	4.96E-05			
SNP-4.16987686.	4	17159646	5.09E-05			
SNP-8.22690109.	8	22692823	5.80E-05			
SNP-9.9087401.	9	9088403	6.09E-05	clr9/qLTG-9	Oh et al. 2004/ Li et al. 2013	LTGP/*ALL*
SNP-10.15677128.	10	15748359	6.23E-05	qCTSS-10	Yang et al. 2013	
SNP-10.11494085.	10	11565267	6.46E-05	qLTG-10	Jiang et al. 2006	
SNP-9.22541472.	9	22541954	6.82E-05			
SNP-3.3410668.	3	3411673	7.15E-05			
SNP-11.3673951.	11	3678050	8.55E-05	qLVG11	Han et al. 2006	
SNP-1.21971200.	1	21972246	8.60E-05			
SNP-7.22920799.	7	22921793	8.63E-05			
SNP-5.15846104.	5	15903564	9.27E-05	qLVG5	Han et al. 2006	
SNP-7.28369125.	7	28370118	9.46E-05	qLVG7-2	Han et al. 2006	
SNP-2.10198335.	2	10198338	9.81E-05			
SNP-1.42375726.	1	42376771	9.96E-05			
SNP-5.15846104.	5	15903564	3.54E-05	qLVG5	Han et al. 2006	
SNP-9.22542045.	9	22542527	5.01E-05			LTGI/*INDICA*
SNP-1.10721450.	1	10722475	7.53E-05			
SNP-7.26278120.	7	26279115	7.76E-05	qLVG7-2	Han et al. 2006	
SNP-11.12306350.	11	12770030	3.20E-05	qLTG-11-1/qSCT11	Jiang et al. 2006/Kim et al. 2014	
SNP-7.26579710.	7	26580705	4.32E-05	qLVG7-2	Han et al. 2006	LTGI/*JAPONICA*
SNP-5.7231674.	5	7231733	6.92E-05			
SNP-11.1093268.	11	1094268	6.51E-05			
SNP-7.9177807.	7	9178802	6.56E-05			LTGP/*INDICA*
SNP-9.22542045.	9	22542527	7.10E-05			
SNP-3.3410668.	3	3411673	7.75E-05			
SNP-2.17728086.	2	17733957	1.15E-05	*qCTS2*		
SNP-11.12306350.	11	12770030	1.15E-05	qLTG-11-1/qSCT11	Jiang et al. 2006/Kim et al. 2014	
SNP-7.22916319.	7	22917313	5.94E-05	qLVG7-2	Han et al. 2006	
SNP-1.31936826.	1	31937871	7.00E-05	*qCTSS-1*		LTGP/*JAPONICA*
SNP-12.17348503.	12	17354549	7.50E-05	*qGR-12*		
SNP-7.8622202.	7	8623198	8.70E-05			
SNP-5.22739346.	5	22801905	9.24E-05	*qLTG-5-2*		

id11006306	11	17467775	1.24E-05	qLTG-11-1/qSCT11	Jiang et al., 2006/Kim et al., 2014	LTGP,44k
id7003987	7	22881924	7.04E-05	qLVG7-2	Han et al.,2006	LTGP,44k
id9007765	9	22531470	5.66E-05			LTGI,44k
id8000984	8	3108387	6.39E-05			
id2007618	2	19505884	8.88E-05	qCTS2	Liu et al., 2013	

Supplementary Table S6. Different SNPs associated with LTG

SNP	Chromosme	position	p-value	QTL	reference
SNP-2.489904.	2	489905	4.20E-06		
SNP-11.12306350.	11	12770030	7.83E-06	qLTG-11-1/qSCT11	Jiang et al. 2006/Kim et al. 2014
SNP-12.6717912.	12	6719023	2.71E-05		
SNP-12.25114399.	12	25147946	3.35E-05		
SNP-7.22916319.	7	22917313	4.44E-05	qLVG7-2	Han et al. 2006
SNP-7.1026437.	7	1027438	4.61E-05		
SNP-1.27481758.	1	27482803	4.85E-05		
SNP-9.22542045.	9	22542527	4.96E-05		
SNP-4.16987686.	4	17159646	5.09E-05		
SNP-8.22690109.	8	22692823	5.80E-05	qSV8-1	Zhang et al. 2005
SNP-9.9087401.	9	9088403	6.09E-05	clr9/qLTG-9	Oh et al. 2004/ Li et al. 2013
SNP-10.15677128.	10	15748359	6.23E-05	qCTSS-10	Yang et al. 2013
SNP-10.11494085.	10	11565267	6.46E-05	qLTG-10	Jiang et al. 2006
SNP-9.22541472.	9	22541954	6.82E-05		
SNP-3.3410668.	3	3411673	7.15E-05		
SNP-11.3673951.	11	3678050	8.55E-05	qLVG11	Han et al. 2006
SNP-1.21971200.	1	21972246	8.60E-05		
SNP-7.22920799.	7	22921793	8.63E-05		
SNP-5.15846104.	5	15903564	9.27E-05	qLVG5	Han et al. 2006
SNP-7.28369125.	7	28370118	9.46E-05	qLVG7-2	Han et al. 2006
SNP-2.10198335.	2	10198338	9.81E-05		
SNP-1.42375726.	1	42376771	9.96E-05		
SNP-1.30553988.	1	30555033	1.30E-05	qLVG-1	Chen et al. 2006
SNP-1.10721450.	1	10722475	1.98E-05		
SNP-2.7783346.	2	7783348	3.81E-05		
SNP-8.3108386.	8	3109384	3.97E-05		
SNP-4.20466240.	4	20638198	4.01E-05		
SNP-8.3114101.	8	3115099	4.40E-05		
SNP-1.30416673.	1	30417718	4.68E-05	qLTG-1	Chen et al. 2006
SNP-7.26278120.	7	26279115	4.93E-05	qLVG7-2	Han et al. 2006
SNP-8.3097042.	8	3098040	6.23E-05		
SNP-3.24464619.	3	24466541	6.37E-05		
SNP-5.16324989.	5	16382508	7.01E-05		
SNP-2.19497594.	2	19503463	7.13E-05		
SNP-11.4219309.	11	4223408	7.27E-05	qLVG11	Han et al. 2006
SNP-9.22844340.	9	22844822	7.34E-05		
SNP-1.23843134.	1	23844179	7.38E-05		
SNP-9.22541111.	9	22541593	7.96E-05		
SNP-9.22512729.	9	22513211	8.39E-05		
SNP-5.20140745.	5	20203273	8.87E-05		
SNP-12.22744562.	12	22778124	9.14E-05		
SNP-11.6595377.	11	6599625	9.25E-05		
SNP-11.16013664.	11	16478788	9.44E-05		
SNP-7.26579710.	7	26580705	4.32E-05	qLVG7-2	Han et al. 2006
SNP-5.7231674.	5	7231733	6.92E-05		
SNP-2.17728086.	2	17733957	1.15E-05	*qCTS2*	
SNP-1.31936826.	1	31937871	7.00E-05	*qCTSS-1*	
SNP-12.17348503.	12	17354549	7.50E-05	*qGR-12*	
id11006306	11	17467775	1.24E-05	qLTG-11-1/qSCT11	Jiang et al., 2006/Kim et al., 2014
id7003987	7	22881924	7.04E-05	qLVG7-2	Han et al.,2006
id9007765	9	22531470	5.66E-05		
id8000984	8	3108387	6.39E-05		
id2007618	2	19505884	8.88E-05	qCTS2	Liu et al., 2013

Supplemental Table 7. Information for the primers used in this study.

**Primer name**	**Sequence (5' to 3')**	**Comment**
OsSAP16qRT-F	CAAAGAACACTGCCTGAAGCATAGA	qRT-PCR
OsSAP16qRT-R	ACTCGTTGCTGCGGTAAGAAGAC	
OsSAP16smRT-F	AAACATCACCTGGGACACCC	Semi-quantitative RT-PCR
OsSAP16smRT-R	ACATCGACCGTCACTCTGCTA	
sap1-LP	CGTCTTCGTAACGGTCTCCC	Genotype
sap1-RP	TGGATCACTGTCCTAAAGGCTAAA	
sap1-LB	ATAGGGTTTCGCTCATGTGTTGAGCAT	
sap2-LP	CCTTTCGGTGATGCTCTTG	Genotype
sap2-RP	TTCTGGATGGCTTGGGTAG	
sap2-LB	AATCCAGATCCCCCGAATTA	
sap3-LP	ACAATGGCTTGCCTTGAAAC	Genotype
sap3-RP	AGGCTGCATATATCCGGTTG	
sap3-LB	CCACAGTTTTCGCGATCCAGACTG	

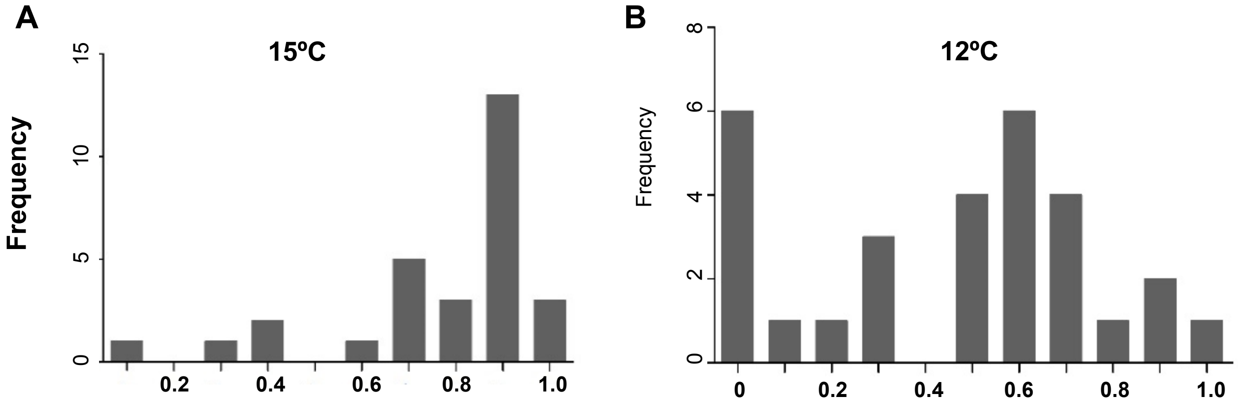

Supplemental Figure S1. Frequency distribution of LTGP of randomly selected 30 accessions under 15ºC (A) and 12ºC (B).

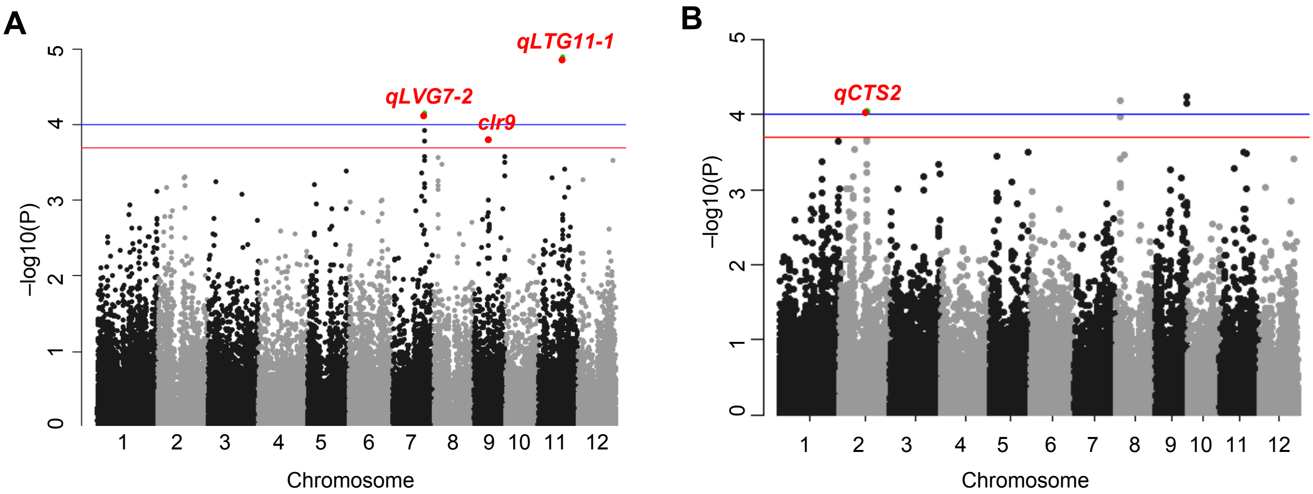

Supplemental Figure S2. Manhattan plots of GWAS on LTGP and LTGI using the 44k dataset. Manhattan plots of LTGP (A) and LTGI (B). Red points represent the SNPs located in the reported QTLs. The blue line indicates the significant threshold set at $p=1.0×10^{-4}$ and the red line represents the minor significance threshold with $p= 2×10^{-4}$.

Plant tissue	Relative expression
Embryo_Coleoptile_Dry_Seed	0.4526
Embryo_Epiblast_Dry_Seed	0.0772
Embryo_Plumule_Dry_Seed	0.5431
Embryo_Radicle_Dry_Seed	0.4402
Embryo_Scutellum_Dry_Seed	0.2946
Embryo_Coleoptile_12hr_post_imbibition	0.1691
Embryo_Epiblast_12hr_post_imbibition	0.3453
Embryo_Plumule_12hr_post_imbibition	0.5328
Embryo_Radicle_12hr_post_imbibition	-0.1049
Embryo_Scutellum_12hr_post_imbibition	0.0342
Embryo_Coleoptile_24hr_post_imbibition	0.2982
Embryo_Epiblast_24hr_post_imbibition	0.2584
Embryo_Plumule_24hr_post_imbibition	0.8629
Embryo_Radicle_24hr_post_imbibition	0.3483
Embryo_Scutellum_24hr_post_imbibition	0.5664
Seedling_Root_tip_Lateral_root_cap	0.5775
Seedling_Root_tip_Cortex	0.275
Seedling_Root_tip_Vascular_bundle	0.5497
Seedling_Root_tip_Central_metaxylem_precursor	0.7937
Seedling_Root_elongation_zone_Central_metaxylem	0.2851
Seedling_Root_elongation_zone_Stele_Vascular_bundle	-0.5925
Seedling_Root_elongation_zone_Cortex	0.3403
Seedling_Root_elongation_zone_Endodermis	0.3317
Seedling_Root_elongation_zone_Epidermis	0.1395
Seedling_Root_maturation_zone_Stele-Vascular_bundle	0.5511
Seedling_Root_maturation_zone_Cortex	0.6336
Seedling_Root_maturation_zone_Endodermis	0.7984
Seedling_Root_maturation_zone_Epidermis	0.1248
Seedling_Shoot_Apical_meristem	0.6212
Seedling_Shoot_P1	-0.042
Seedling_Shoot_P2	0.7729
Seedling_Shoot_P3	1.0943
Seedling_Shoot_Axillary_meristem	1.4484
Seedling_Shoot_Axillary_primordium	0.6385
Seedling_Leaf_blade_2nd_leaf_Mesophyll	0.9619
Seedling_Leaf_blade_2nd_leaf_Bundle_sheath	-0.3534
Seedling_Leaf_blade_2nd_leaf_Vein	0.5669
Seedling_Leaf_blade_2nd_leaf_Bulliform	0.4254
Seedling_Leaf_blade_2nd_leaf_Epidermal_Long_Cell	0.312
Seedling_Leaf_blade_2nd_leaf_Stomata	0.7114
Tissue_Whole_root_fresh	-0.0825
Tissue_Whole_leaf_blade_fresh	0.2312

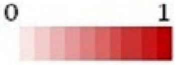

Supplemental Figure S3. Relative expression of *OsSAP16* in different cell types and plant tissues of rice. Data is captured from ROAD (http://www.ricearray.org/).

Dataset: 760 perturbations from data selection: OS_AFFY_RICE-1

Showing 1 measure(s) of 1 gene(s) on selection: OS-0

Log2-ratio

-2.5 -2.0 -1.5 -1.0 -0.5 0.0 0.5 1.0 1.5 2.0 2.5

Down-regulated Up-regulated

760 of 760 perturbations fulfilled the filter criteria

Filter values for OS07G0569700

Oryza sativa (760)

	OS07G0569700 Log2-ratio (no filter)	Fold-Change (no filter)	p-value (no filter)
germination (4h) / embryo samples from dry seeds (Nipponbare)	1.75	3.36	<0.001
diurnal study 2 (4:00pm day 2) / diurnal study 2 (8:00am day 2)	1.72	3.28	0.005
germination (24h) / embryo samples from dry seeds (Nipponbare)	1.64	3.10	0.001
germination (16h) / embryo samples from dry seeds (Nipponbare)	1.63	3.08	0.001
anaerobic germination (3h) / anaerobic germination (1h)	1.58	2.91	0.003
cold study 10 (IR29) / untreated leaf samples (IR29)	1.45	2.82	0.005
germination (12h) / embryo samples from dry seeds (Nipponbare)	1.45	2.73	0.003
cold study 10 (LTH) / untreated leaf samples (LTH)	1.41	2.68	<0.001
cold study 2 (Huahui 1) / untreated leaf samples (Huahui 1)	1.35	2.55	<0.001
non-aromatic RILs / Pusa 1342	1.30	2.48	<0.001
heat study 2 (Huahui 1) / untreated leaf samples (Huahui 1)	1.28	2.42	<0.001
germination (8h) / embryo samples from dry seeds (Nipponbare)	1.27	2.41	0.002
cold study 2 (MH63) / untreated leaf samples (MH63)	1.27	2.41	<0.001
aerobic germination study 2 (3h) / embryo samples from dry seeds (Amaroo)	1.24	2.37	0.001
A. tumefaciens (Nipponbare) / untreated calli samples (Nipponbare)	1.23	2.34	0.036
heat study 2 (MH63) / untreated leaf samples (MH63)	1.20	2.30	<0.001
diurnal study 2 (4:00pm day 1) / diurnal study 2 (8:00am day 1)	1.14	2.17	0.055
PR 319 / PMS 80	1.12	2.15	0.002
aerobic germination study 2 (24h) / embryo samples from dry seeds (Amaroo)	1.10	2.14	<0.001
A. tumefaciens study 2 (Nipponbare) / untreated calli samples (Nipponbare)	1.06	2.06	0.044
diurnal study 2 (12:00am day 1) / diurnal study 2 (8:00am day 1)	1.00	1.97	0.071
A. tumefaciens (6h) / untreated calli samples	0.97	1.95	0.009
A. tumefaciens (1h) / untreated calli samples	0.96	1.95	0.010
X. oryzae pv. oryzae T7174 (Nipponbare) / mock treated leaf samples (Nipponbare)	0.96	1.97	0.006
cold study 11 (LTH) / untreated leaf samples (LTH)	0.95	1.93	<0.001
non-aromatic RILs / Pusa 1121	0.91	1.86	0.001
Pusa 1266 / Pusa Basmati 1	0.90	1.87	0.001
aerobic germination study 2 (12h) / embryo samples from dry seeds (Amaroo)	0.89	1.86	<0.001
M. grisea (4dpi) / mock treated leaf samples (4dpi)	0.89	1.85	0.006
cold study 11 (IR29) / untreated leaf samples (IR29)	0.89	1.85	<0.001
spl5 / Zhefu802	0.88	1.84	0.034
MRP 5401 / PMS 80	0.87	1.82	<0.001
cold study 12 (LTH) / untreated leaf samples (LTH)	0.85	1.82	0.002

Supplemental Figure S4. Gene expression profiles of *OsSAP16* from GENEVESTIGATOR (https://en.wikipedia.org/wiki/Genevestigator).

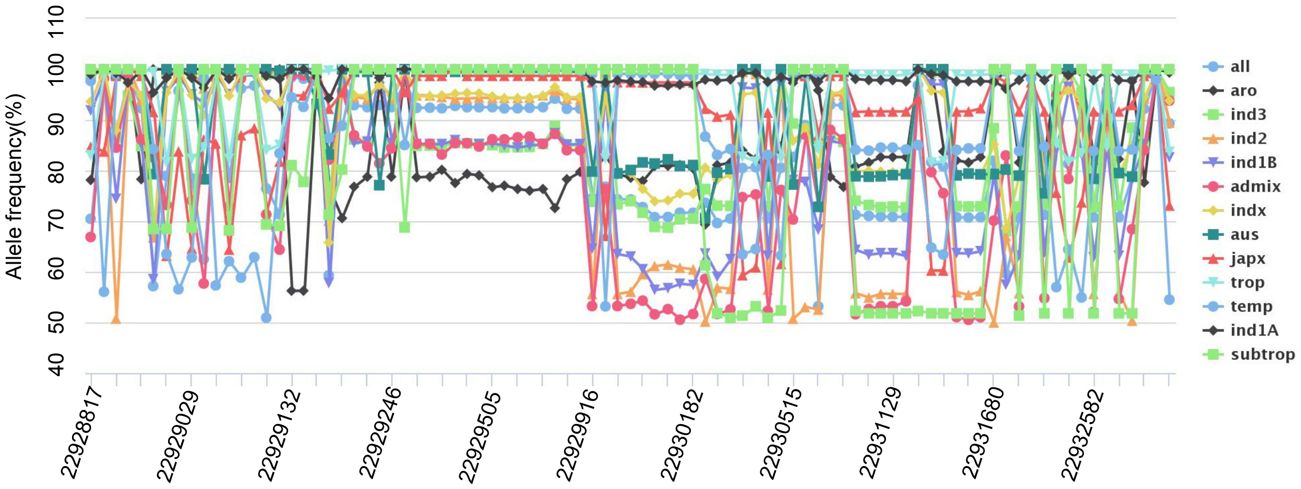

Supplemental Figure S5. SNPs located in *OsSAP16* across the 2945 rice varieties based on IRIC (http://www.oryzasnp.org/).

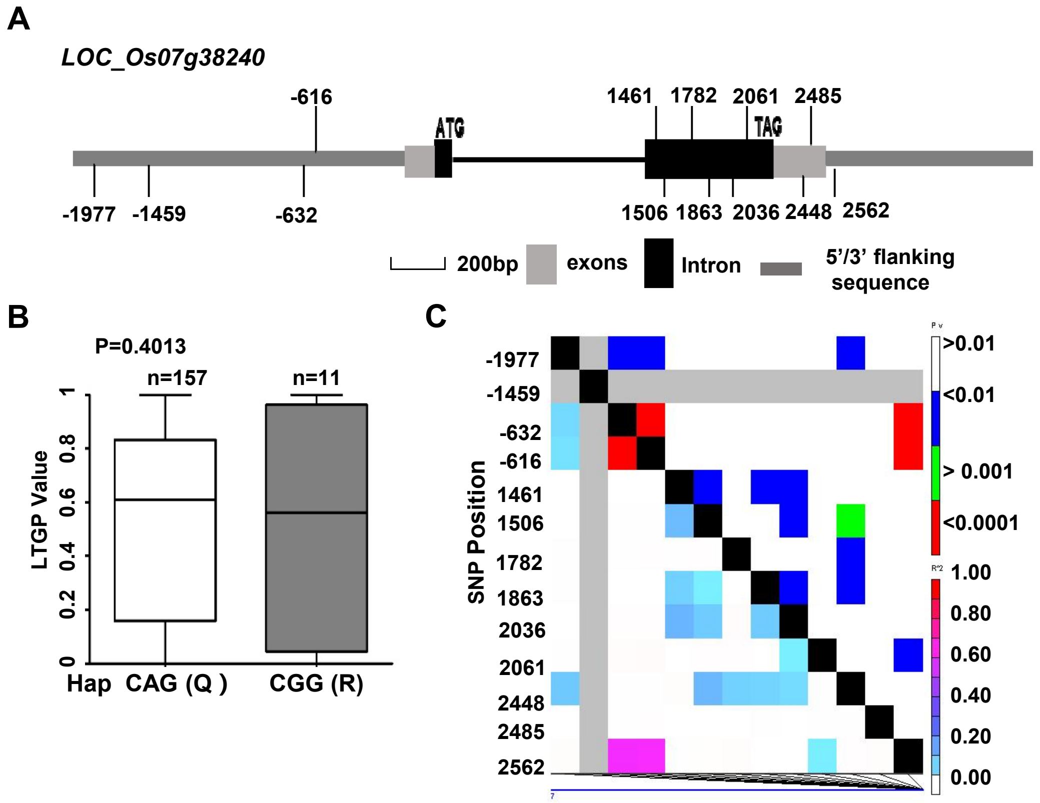

Supplemental Figure S6. SNPs of the *OsSAP16* gene present in the 700k dataset.
A, DNA polymorphism in the *OsSAP16* gene. Position is defined relative to the transcription start site.
B, Boxplot of LTGI based on the two haplotypes at the 2036 bp 5' to the transcription start site. A is part of CAG codon for phenylalanine and G is part of the CGG codon for Arginine.
C, LD plot of the 13 SNPs in *OsSAP16*. Upper triangle is P-Value and lower triangle is R squared.

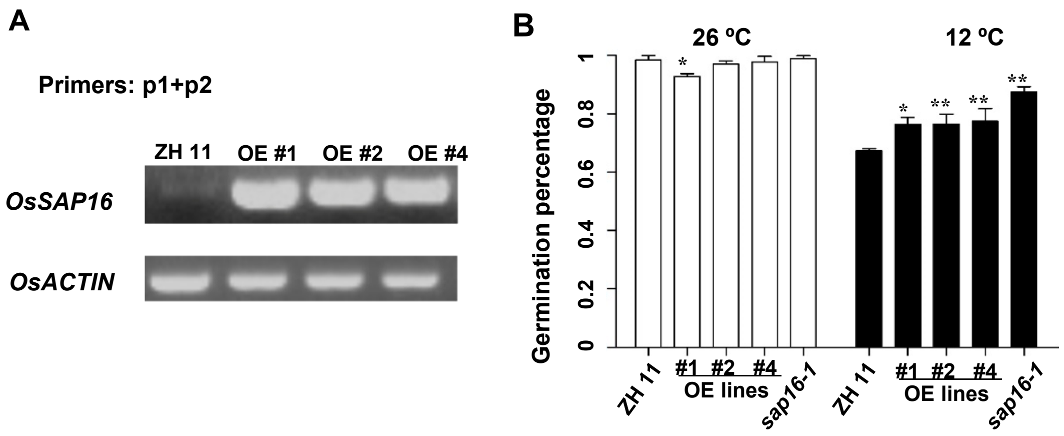

Supplemental Figure S7. Phenotypes of *OsSAP16* overexpression lines.

A, Semi-quantitative RT-PCR analysis of *OsSAP16* expression in overexpression lines. The *OsACTIN* gene was used as a normalization control. Shown are products from 28 cycles of amplification for *OsACTIN* and *OsSAP16* in all samples.

B, Germinability of wild-type Zhonghua11 (ZH11), OE lines, and *sap16-1* under low (12ºC) and normal (26ºC) temperature growth condition. Seeds incubated for 3 days under 26ºC and 13 days under 12ºC. All data in the graph are means ± s.d. from three replicates. Differences of mutants compared to wild types were analyzed by Welch's t-test. $^{*}P < 0.05$, $^{**}P < 0.01$.

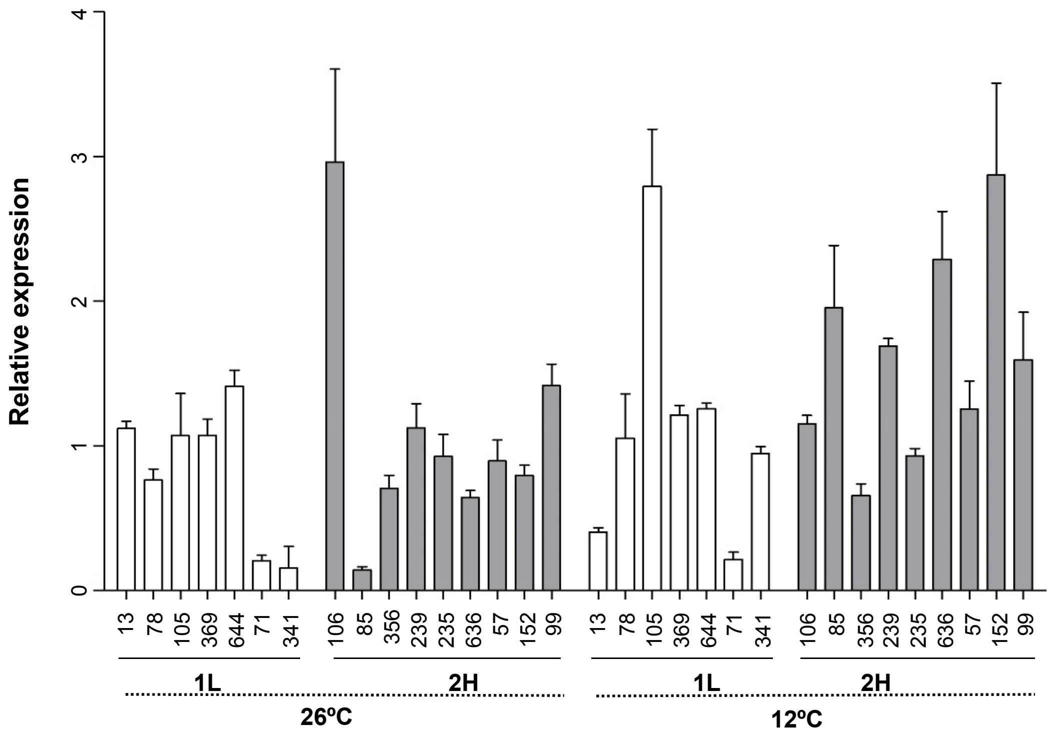

Supplemental Figure S8. Relative expressions of *OsSAP16* in imbibed seeds of various accessions after two days in dark at 12ºC and 26ºC respectively.

1H represents Hap1 with high LTG, 2L represents Hap2 with low LTG, 1L represents Hap1 with low LTG, 2H represents Hap2 with high LTG. Only the number part of the accession name of RDP1 is shown, e.g., 13 represents NSFTV_13. All data in the graph are means ± s.d. from three replicates.

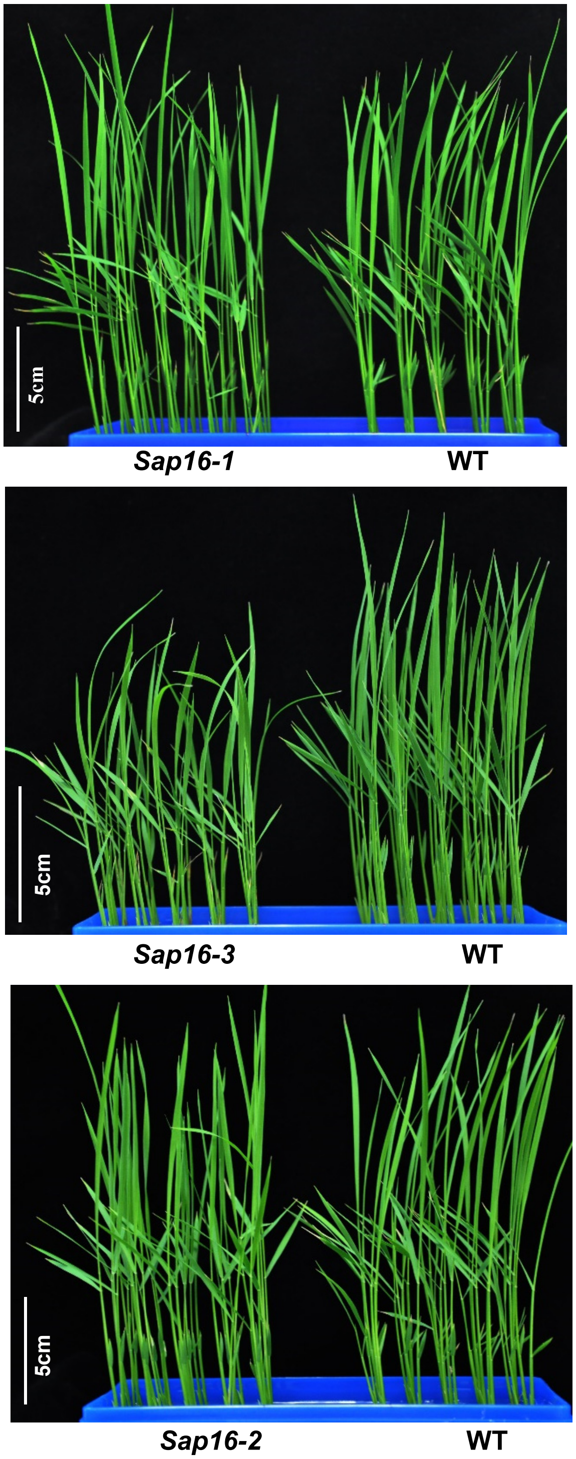

Supplemental Figure S9. *OsSAP16* mutants and wild type (WT) grown at 26ºC for 14 days in 0.35% agar.
